# Identification of embolic stroke in patients with large vessel occlusion: The Chinese embolic stroke score, CHESS

**DOI:** 10.1111/cns.13729

**Published:** 2021-09-24

**Authors:** Lan Hong, Longting Lin, Gang Li, Jianhong Yang, Yu Geng, Min Lou, Mark Parsons, Xin Cheng, Qiang Dong

**Affiliations:** ^1^ Department of Neurology National Center for Neurological Disorders, National Clinical Research Centre for Aging and Medicine, Huashan Hospital, State Key Laboratory of Medical Neurobiology, Fudan University Shanghai China; ^2^ South Western Sydney Clinical School University of New South Wales Liverpool Australia; ^3^ Department of Neurology Shanghai East Hospital Tongji University Shanghai China; ^4^ Department of Neurology Ningbo First Hospital Ningbo China; ^5^ Department of Neurology Zhejiang Provincial People’s Hospital People’s Hospital of Hangzhou Medical College Hangzhou China; ^6^ Department of Neurology the Second Affiliated Hospital of Zhejiang University Hangzhou China

**Keywords:** angioplasty, embolic stroke, perfusion imaging, thrombectomy

## Abstract

**Aims:**

The aim of the study was to develop a simple and objective score using clinical variables and quantified perfusion measures to identify embolic stroke with large vessel occlusions.

**Methods:**

Eligible patients from five centers participating in the International Stroke Perfusion Imaging Registry were included in this study. Patients were split into a derivation cohort (*n* = 213) and a validation cohort (*n* = 116). A score was developed according to the coefficients of independent predictors of embolic stroke from stepwise logistic regression model in the derivation cohort. The performance of the score was validated by assessing its discrimination and calibration.

**Results:**

The independent predictors of embolic stroke made up the Chinese Embolic Stroke Score (CHESS). There were: history of atrial fibrillation (3 points), non‐hypertension history (2 points), and delay time>6 s volume/delay time>3 s volume on perfusion imaging ≥0.23 (2 points). The AUC of CHESS in the derivation cohort and validation cohort were 0.87 and 0.79, respectively. Patients with a CHESS of 0 could be identified as low‐risk of embolic stroke, with a CHESS of 2–4 could be identified as medium‐risk and with a CHESS of 5–7 could be regarded as high‐risk. The observed rate of embolic stroke of each risk group was well‐calibrated with the predicted rate.

**Conclusion:**

CHESS could reliably and independently identify embolic stroke as the cause of large vessel occlusion.

## 1 INTRODUCTION

Endovascular treatment has been globally acknowledged as a standard‐of‐care for acute ischemic stroke patients with large vessel occlusion (LVO), and prompt reperfusion is a key to achieving a satisfactory clinical outcome. However, Chinese LVO patients have been faced with the challenge of a longer reperfusion time compared with the Western population.^1^, ^2^, ^3^, ^4^, ^5^ One of the main reasons may relate to the high prevalence of in‐situ thrombosis due to intracranial atherosclerotic disease (ICAD) in Asian patients with acute LVO,^6^ which is refractory to the current stent retrievers or aspiration systems and requires rescue therapy, like angioplasty and stenting.^7^ Additionally, the mechanism of embolic stroke can also be very complicated due to cardiogenic embolism, artery to artery embolism from large vessel atherosclerosis and embolic stroke of undetermined source, which requires different recanalization techniques. Thus, precisely identifying the etiology of the occlusion (embolic or non‐embolic) at hyperacute stage before reperfusion therapy is crucial to the choice of the optimal recanalization strategy.

Several imaging markers have been developed to identify stroke etiology using thrombus location and clot formation.^8^, ^9^, ^10^, ^11^, ^12^, ^13^, ^14^, ^15^ Although they have a good predictive ability, the accuracy of these imaging markers depends on readers’ experience, confining its application under emergent settings. Another marker of embolic versus ICAD as the cause of LVO is that ICAD patients have a better collateral flow,^16^ which could quantitively assessed by perfusion imaging.^17^, ^18^ We aimed to develop and validate a concise and objective score combining baseline clinical data and a quantified measure of collateral flow using perfusion imaging to distinguish between embolic and non‐embolic stroke preceding endovascular treatment. Additionally, though history of atrial fibrillation (AF) is a strong predictor of embolic stroke, it may not be easily known under emergent conditions. Moreover, acute LVO due to in‐situ thrombosis may also occur in patients with AF. Therefore, the performance of the score that excluded history of AF was also assessed in the derivation and validation cohort.

## 2 METHODS

This study adheres to the Transparent Reporting of a Multivariable Prediction Model for Individual Prognosis or Diagnosis (TRIPOD) statement.

### 2.1 Patient selection and definition of embolic stroke

Consecutive acute ischemic stroke patients presenting within 24 h of symptom onset at the five sites participating in the International Stroke Perfusion Imaging Registry (INSPIRE) from 2015 to 2019 were included. Patients were eligible for the current study if they (1) underwent complete baseline multimodal CT imaging, including non‐contrast computed tomography (CT), CT Perfusion (CTP), CT Angiography (CTA); (2) had large vessel occlusion/severe stenosis; (3) had complete baseline clinical profiles; (4) underwent endovascular treatment. CTA imaging was acquired from aorta arc to vertex. Severe stenosis was defined as >50% stenosis of the vessel caliber of the ipsilateral large artery in CTA using the diameter of the adjacent contact segment as the reference. Baseline National Institutes of Health Stroke Scale (NIHSS) was assessed by the stroke neurologist arrived in the Emergency Room. The BP measured in the emergency room (ER) for the first time after arrival was considered baseline. Previous medical history including history of hypertension, history of diabetes mellitus, history of atrial fibrillation, history of dyslipidemia, history of smoking and past history of stroke was provided by the patients and their relatives. History of smoking was identified as any previous history of smoking. All patients underwent emergent electrocardiograms, 24 h Holter monitoring, and transthoracic echocardiography in hospital in order to help identify stroke etiology according to Trial of ORG 10172 in Acute Stroke Treatment (TOAST) classification.^19^ Written informed consent was obtained from each participant as per local approvals. The study was approved by local ethics committees.

An embolic stroke was determined using digital subtraction angiography imaging (DSA). Patients with a DSA imaging where there was no residual severe stenosis (>50% stenosis of the vessel caliber, using the diameter of the adjacent contact segment as the reference) after thrombectomy (without angioplasty or stenting) in the culprit occluded artery was considered embolic. DSA imaging was centrally and retrospectively analyzed by Dr. Lin and Dr. Hong blind to the CT Perfusion parameters and clinical information. Patients who underwent direct angioplasty were classified as non‐embolic stroke. When discrepancy occurred between the two investigators, a third investigator (Dr. Cheng) was assigned to determine the cause of this index LVO.

### 2.2 Acute multimodal imaging protocol and perfusion imaging analysis

Patients from these five Chinese sites were scanned using 64‐, 256‐, or 320‐slice detector scanners for non‐contrast CT, CT Angiography, and CT Perfusion. Details of different CT scanners are provided in Table S1.

**TABLE 1:** Comparisons of baseline clinical profiles and CTP data in the derivation and validation cohort^†^

	Derivation Cohort			Validation Cohort		
	Non‐Embolic (*n* = 60)	Embolic Stroke (*n* = 153)	*p*	Non‐Embolic (*n* = 33)	Embolic Stroke (*n* = 83)	*p*
Age, median (IQR), years	63.0 (54.0, 73.8)	70.0 (64.5, 78.0)	0.001	70.0 (59.5, 80.5)	73.0 (64.0, 82.0)	0.43
Male	44 (73.3%)	84 (54.9%)	0.002	26 (78.8%)	45 (54.2%)	0.01
Baseline SBP, median (IQR)/ mean (SD), mmHg	150.0 (139.3, 163.0)	147.0 (128.0, 163.0)	0.25	147.7 (27.6)	103.5 (14.7)	0.90
Baseline DBP, mean (SD)/ median (IQR), mmHg	87.2 (13.8)	81.3 (12.1)	0.01	84.0 (76.0, 97.5)	80.0 (74.0, 90.0)	0.15
Baseline Glucose, median (IQR), mmol/L	7.0 (6.1, 8.8)	7.1 (6.4, 8.9)	0.58	7.5 (5.9, 9.6)	7.1 (6.0, 9.2)	0.89
Baseline NIHSS, median (IQR)	13.0 (10.0, 19.0)	17.0 (13.0, 20.5)	0.003	15.0 (9.5, 18.0)	17.0 (12.0, 20.0)	0.05
Medical History						
History of Smoking	27 (45.0%)	36 (23.5%)	0.002	14 (42.4%)	18 (21.7%)	0.02
History of Hypertension	44 (73.3%)	91 (59.5%)	0.06	25 (75.8%)	47 (56.6%)	0.06
History of Atrial Fibrillation	8 (13.3%)	104 (68.0%)	<0.001	5 (15.2%	51 (61.5%)	<0.001
History of Dyslipidemia	10 (16.7%)	19 (12.4%)	0.24	1 (3.0%)	10 (12.1%)	0.29
History of Diabetes Mellitus	14 (23.3%)	25 (16.3%)	0.24	16 (48.5%)	14 (16.9%)	<0.001
Past History of Stroke or TIA	13 (21.7%)	18 (11.8%)	0.07	4 (12.1%)	15 (18.1%)	0.44
Cause of Stroke			<0.001			<0.001
Large Artery Atherosclerosis	55 (91.7%)	14 (9.2%)		30 (90.9%)	9 (10.8%)	
Cardiac Embolism	0 (0.0%)	113 (73.9%)		0 (0.0%)	57 (68.7%)	
Others^‡^	5 (8.3%)	26 (17.0%)		3 (9.1%)	17 (20.5%)	
Occlusion Site			0.80			0.93
ICA	13 (21.7%)	38 (24.8%)		9 (27.3%)	17 (20.5%)	
MCA‐M1	32 (53.3%)	81 (52.9%)		16 (48.5%)	43 (51.8%)	
MCA‐M2	1 (1.7%)	7 (4.6%)		1 (3.0%)	4 (4.8%)	
ICA+MCA‐M1	4 (6.7%)	9 (5.9%)		2 (6.1%)	8 (9.6%)	
ACA	0 (0.0%)	1 (0.7%)		0 (0.0%)	1 (1.2%)	
PCA/VA/BA	10 (16.7%)	17 (11.1%)		5 (15.2%)	10 (12.0%)	
Onset to door time, median (IQR), min	206.0 (118.0, 345.0)	180.0 (99.5, 280.5)	0.26	192.0 (114.5, 576.3)	176.0 (71.0, 288.0)	0.23
Infarct core, median (IQR), ml	8.0 (2.0, 28.3)	23.0 (8.0, 48.5)	<0.001	10.0 (0.5, 35.5)	15.0 (7.0, 43.0)	0.06
Penumbra, median (IQR), ml	71.5 (41.0, 117.8)	85.0 (60.5, 113.0)	0.19	68.0 (31.0, 152.5)	87.7 (56.0, 117.0)	0.59
DT>3 s, median (IQR), ml	94.0 (54.5, 132.8)	117.0 (78.5, 156.0)	0.02	69.0 (35.5, 188.0)	111.0 (82.0, 149.0)	0.31
DT>6 s, median (IQR), ml	12.5 (3.0, 47.0)	44.0 (12.3, 71.5)	<0.001	17.0 (0.0, 65.5)	45.0 (17.0,75.0)	0.05
DT6/DT3 ratio, median (IQR)	0.2 (0.1, 0.3)	0.4 (0.2, 0.5)	<0.001	0.2 (0.0, 0.4)	0.4 (0.2, 0.5)	0.01

^†^ Data are presented as number (percentage) of patients unless otherwise indicated.

^‡^ Other causes of embolic stroke included embolic stroke of undetermined source, hypercoagulation, stroke of undermined causes. Other causes of non‐embolic stroke included dissection of ipsilateral carotid artery, syphilis and hypoperfusion.

Abbreviations: ACA, anterior cerebral artery; BA, basilar artery; DBP, diastolic blood pressure; DT, delay time; ICA, internal carotid artery; IQR, interquartile range; MAP, mean arterial pressure; MCA‐M1, M1 segment of middle cerebral artery; MCA‐M2, M2 segment of middle cerebral artery; NIHSS, National Institutes of Health Stroke Scale; PCA, posterior cerebral artery; SBP, systolic blood pressure; SD, standard deviation; TIA, transient ischemic attack; VA, vertebral artery.

All perfusion images were centrally post‐analyzed using the commercial software MIStar (Apollo Medical Imaging Technology) with single value deconvolution with delay and dispersion correction. Hypoperfusion volume and core volume were calculated using previously validated thresholds (Hypoperfusion lesion: delay time [DT]>3 s, Core: relative cerebral blood flow[rCBF]<30%, Severe hyopoperfused lesion: DT>6 s).^20^, ^21^, ^22^ Acute cerebral collateral flow was quantified using the volume ratio of severely delayed contrast transit tissue (delay time [DT]>6 s) within the DT>3 s perfusion lesion in patients with large vessel occlusions of anterior or posterior circulation.^17^, ^18^

### 2.3 Statistical analysis

Statistical analysis was performed using Stata v15.0 (StataCorp, College Station). Graphs were drawn using Stata v15.0 (StataCorp, College Station) or Prism v8.0 (GraphPad Software). Mean and standard deviation were used to describe continuous variables if normally distributed, or median and interquartile range (IQR) if not normally distributed. Categorical variables were described using percentage. For continuous variables, normality was tested using the Shapiro‐Wilk test. Since the percentage of embolic stroke is much higher than the non‐embolic ones in our cohorts, univariate comparisons of baseline clinical and imaging variables between embolic stroke and non‐embolic stroke, derivation cohort and validation cohort were performed using Welch's *t* test for normally distributed continuous variables, Wilcoxon's rank‐sum test for skewedly distributed continuous variables, and chi‐squared or Fisher's exact test for categorical variables. A two‐tailed *p* < 0.1 was considered significant in the univariate analysis within the derivation cohort in order to acquire potential predictors. And in other steps of statistical analysis, a two‐tailed *p* < 0.05 was considered significant.

The study cohort was divided into derivation and validation cohorts for analysis (65% and 35% respectively) using stratified sampling according to the rate of embolic stroke in the whole cohort using the “stratarand” command in Stata.

#### 2.3.1 Model Derivation

The model was then derived through the following steps: (1) Each statistically significant continuous variable was dichotomized using an optimal cut‐point, which maximized the Youden index. (2) Dichotomized variables were entered into the backward logistic regression model with a stepwise removal probability of *p* < 0.05 to select independent predictors of embolic stoke. (3) The coefficients of independent predictors of embolic stroke were rounded to the nearest integer to generate an integer‐based scoring system: CHinese Embolic Stroke Score (CHESS). The overall score of each patient was calculated as the sum of the variables’ weighted scores if the patient presented any of the selected variables.

#### 2.3.2 Model Validation

The discrimination of the model was tested using the area under the receiver operating characteristic curve (AUC‐ROC) in derivation and validation cohort. The 95% confidence intervals (95%CI) of AUC‐ROCs were also provided. Calibration was evaluated using Hosmer‐Lemeshow goodness‐of‐fit test and was plotted through calibration plots. The AUCs of CHESS in patients with large vessel occlusions of anterior circulation and posterior circulation were also compared. A two‐tailed *p* < 0.05 was considered significant.

## 3 RESULTS

From 2015 to 2019, a total number of 453 patients with LVO who underwent endovascular treatment were recruited in INSPIRE. Among them, one hundred and twenty‐four patients were excluded because of incomplete imaging or clinical data. Therefore, a total number of 329 patients were included in this study.

After the stratified sampling, the derivation cohort included 213 patients, while the validation cohort included the remaining 116 patients. The comparison of baseline demographic, clinical, and imaging profiles between the derivation and validation cohort is listed in Table S2.

**TABLE 2:** CHESS model

Variables	Score Points	Coefficient	OR	95%CI of OR	*p*
History of diagnosed AF	3	3.0	19.5	7.7–49.5	<0.001
No history of hypertension	2	1.7	5.6	2.4–13.6	<0.001
DT>6 s / DT>3 s ≥0.23	2	1.7	5.3	2.4–11.9	<0.001

Abbreviations: 95% CI 95% confidence interval; AF, Atrial fibrillation; CHESS, Chinese Embolic Stroke Score; DT, delay time; NIHSS, National Institutes of Health Stroke Scale; OR odds ratio.

### 3.1 Derivation and internal validation of the score

**FIGURE 1:**
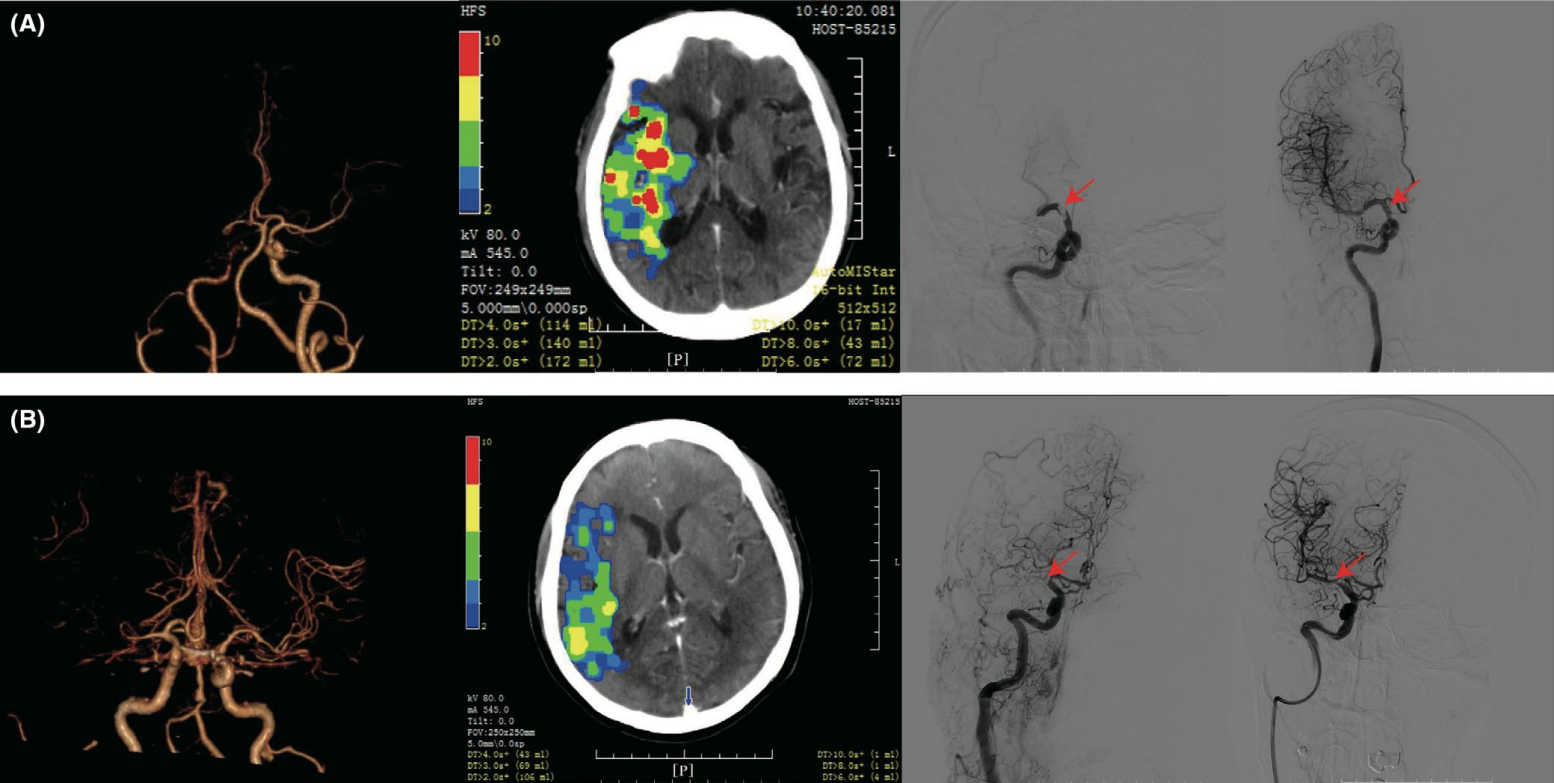
Illustration figures of embolic /non‐embolic stroke patients. A multimodal CT imaging of a patients with embolic stroke: (Left to right) Baseline CTA showed RICA‐IC occlusion; baseline CTP automatically post‐processed by MIStar showed DT>6 s volume of 72 ml and DT>3 s volume of 140 ml (DT>6 s/DT>3 s = 0.51); first angiographic run of DSA imaging showed RICA‐IC occlusion (red arrowhead); angiographic run after pure thrombectomy showed no residual stenosis in the culprit vessel (red arrowhead). B Multimodal CT imaging of a patients with non‐embolic stroke: (Left to right) Baseline CTA showed RMCA‐M1 occlusion; baseline CTP automatically post‐processed by MIStar showed DT>6 s volume of 4 ml and DT>3 s volume of 69 ml (DT>6 s/DT>3 s = 0.06); first angiographic run of DSA imaging showed RMCA‐M1 occlusion (red arrowhead); angiographic run after pure thrombectomy showed severe residual stenosis in the culprit vessel (red arrowhead). Abbreviations: CT—Computed tomography; CTA—CT Angiography; CTP—CT Perfusion; DT—Delay time; DSA—Digital subtraction angiography; RICA‐IC—Intracranial segment of right internal carotid artery; RMCA‐M1—M1‐Segment of right middle cerebral artery

In the derivation cohort, compared with patients with non‐embolic stroke, patients with embolic stroke were older, had lower baseline diastolic blood pressure, higher baseline NIHSS, a more severe perfusion imaging profile, higher rate of AF history and were more frequently female. Fewer patients with embolic stroke had a history of smoking or hypertension (Table 1). In order to avoid collinearity and to acquire a concise and parsimonious prediction model, only one of blood pressure parameters (baseline diastolic blood pressure) and one of the perfusion parameters (DT>6 s/DT>3 s) were entered into the screening model. The optimal cut‐point and Youden index for each statistically significant continuous variables were as follows: Baseline NIHSS, cut‐point 14, Youden index 0.25; age, cut‐point 64 years old, Youden index 0.29; baseline diastolic pressure, cut‐point 113 mmHg, Youden index 0.01; DT>6 s/DT>3 s, cut‐point 0.23, Youden index 0.41. Backward stepwise logistic regression identified 3 independent predictors of embolic stroke: history of AF, history of hypertension and DT>6 s/DT>3 s. The *p*‐values of the excluded variables were as follows: Baseline diastolic pressure≥113 mmHg, *p* = 0.98; age≥64 years old, *p* = 0.65; baseline NIHSS≥14, *p* = 0.16; non‐smoking history, *p* = 0.65; sex, *p* = 0.17; past history of stroke or TIA, *p* = 0.08. The regression coefficients of the variables included in the final model and the points assigned to each variable in the final score are presented in Table 2. The representative multimodal imaging of patients with embolic and non‐embolic stroke has been illustrated in Figure 1.

**FIGURE 2:**
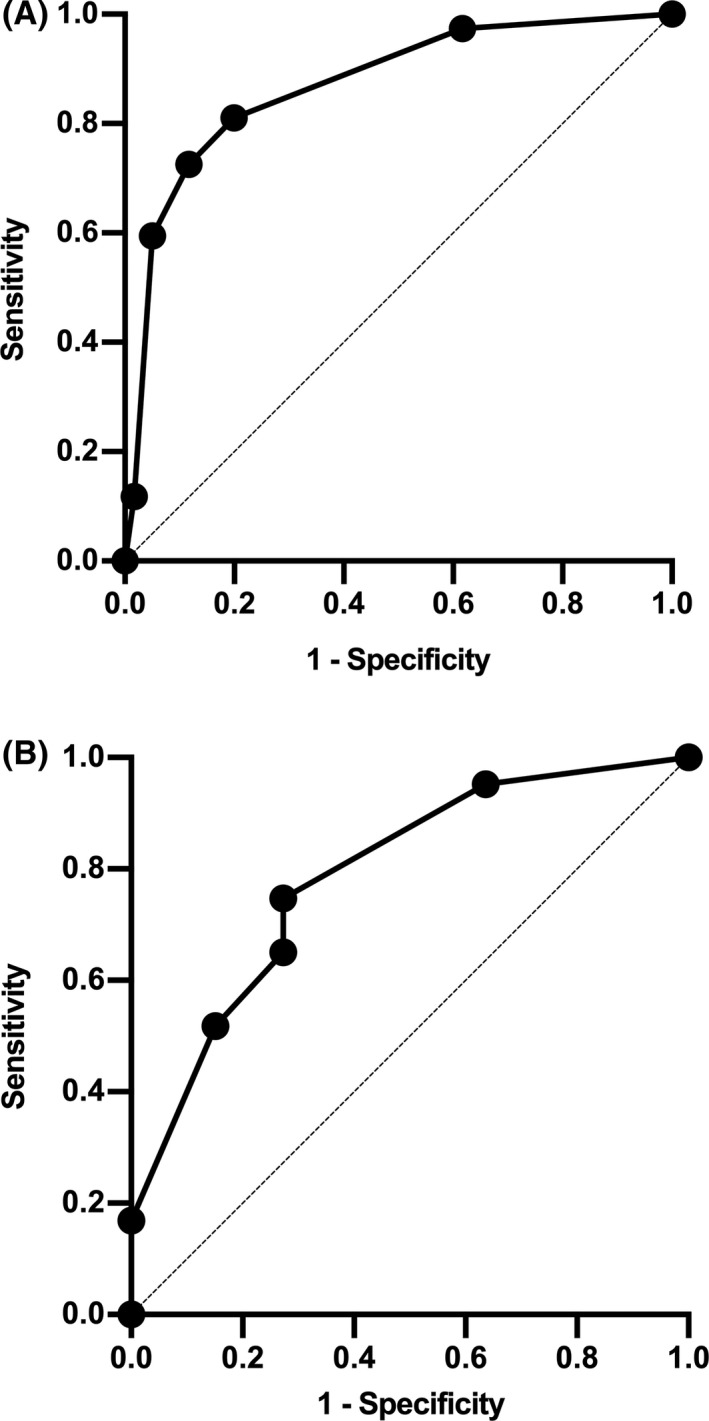
ROC‐AUC of CHESS. A. Derivation cohort: ROC‐AUC of CHESS = 0.87, 95%CI 0.82–0.92. B. Validation cohort: ROC‐AUC of CHESS = 0.79, 95%CI 0.70–0.88. There are 6 dots in the ROC curve (the dot located in the origin of the coordinates is not included for it stands for a theoretical value with 0% sensitivity and 100% specificity), which represent the 6 possible values of CHESS (left to right: 7, 5, 4, 3, 2, 0;CHESS has no score of 1 or 6). Abbreviations: ROC‐AUC Area under the receiver operating characteristic curve; AF Atrial fibrillation; CHESS Chinese Embolic Stroke Score

CHESS exhibited high discriminatory value with an AUC of 0.87 (95%CI 0.82–0.92, Figure 2A). The observed percentages of embolic stroke corresponded well with the predicted possibilities (Hosmer‐Lemeshow goodness‐of‐fit chi‐square = 5.7, *p* = 0.2). No statistically significant difference of AUC of CHESS was found between patients with large vessel occlusion of anterior circulation and posterior circulation (anterior circulation: 0.88 [95% CI 0.82–0.93], posterior circulation 0.86 [95%CI 0.71–1.00], *p* = 0.83). The percentage of patients with each CHESS was as follows: 0, 27 patients (12.7%), 2, 50 patients (23.5%); 3, 18 patients (8.5%); 4, 24 patients (11.3%); 5, 75 patients (35.2%); 7, 19 patients (8.9%).

**FIGURE 3:**
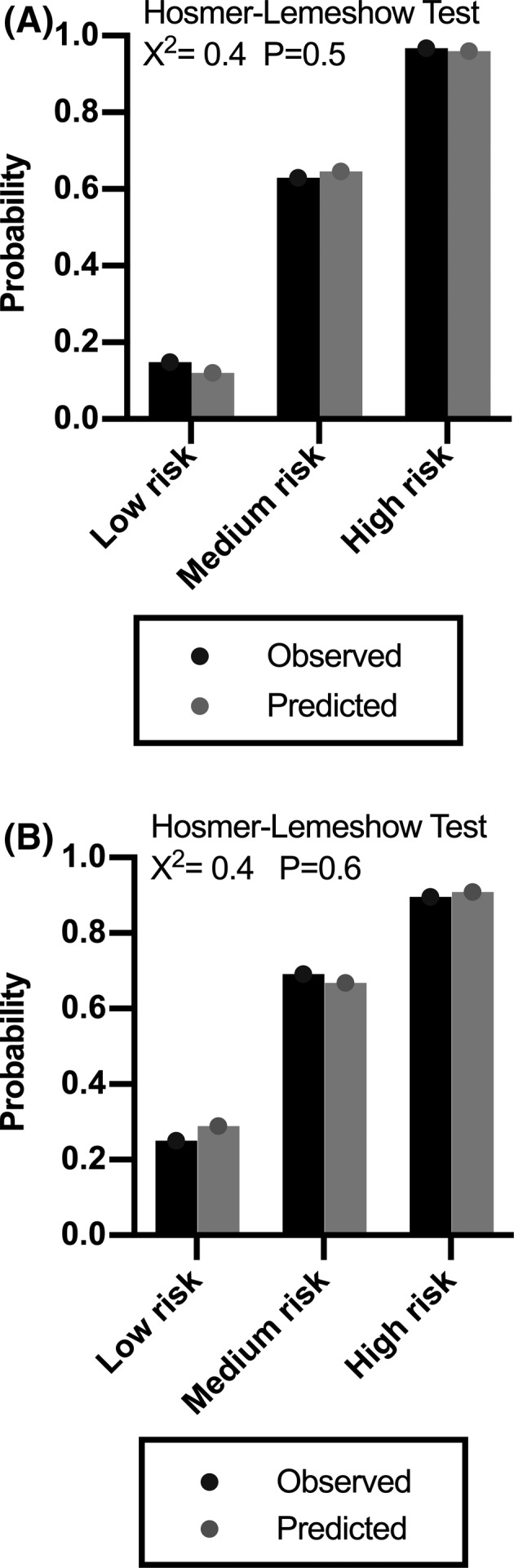
Model calibration of derivation cohort (A) and validation cohort (B) according to different risk level of embolic stroke. Low risk: CHESS 0; Medium risk: CHESS 2–4; High risk: CHESS 5–7. Abbreviations: CHESS Chinese Embolic Stroke Score

**FIGURE 4:**
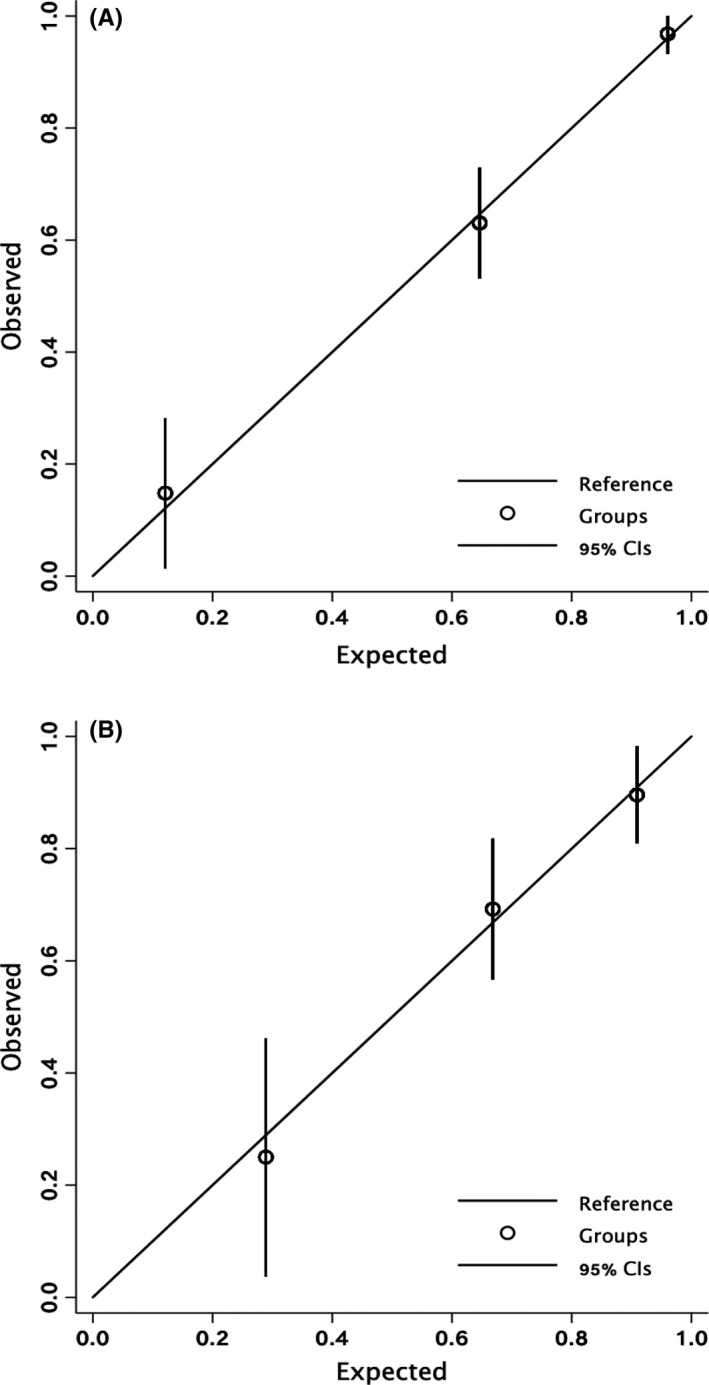
Calibration plot of identifying embolic stroke in derivation cohort (A) and validation cohort (B) according to different risk levels of embolic stroke. Low risk: CHESS 0; Medium risk: CHESS 2–4; High risk: CHESS 5–7. Dots left to right: Low risk, Medium risk, High risk. Every dot locates around the reference line, meaning that the risk stratification of CHESS has a satisfactory calibration to predict embolic stroke. Abbreviations: CHESS Chinese Embolic Stroke Score

Patients were then divided into 3 risk groups of embolic stroke according to their scores (Low risk: CHESS 0, 27 [12.7%] patients; Medium risk: CHESS 2–4, 92 [43.2%] patients); High risk: CHESS 5–7, 94 [44.1%] patients). The observed rates of embolic stroke for low‐, medium‐, and high‐risk group were 14.8%, 63.0%, and 96.8%, respectively, which were well‐calibrated with the predicted rates. (Hosmer‐Lemeshow goodness‐of‐fit chi‐square = 0.4, *p* = 0.5, Figure 3A and Figure 4A).

### 3.2 Model validation

CHESS also performed well in the validation cohort with good discrimination and calibration (AUC = 0.79, 95%CI 0.70–0.88, Figure 2B; Hosmer‐Lemeshow goodness‐of‐fit chi‐square = 5.1, *p* = 0.3). No statistically significant difference of AUC of CHESS was found between patients with large vessel occlusion of anterior circulation and posterior circulation (anterior circulation: 0.79 [95% CI 0.70–0.86], posterior circulation 0.80 [95% CI 0.60−1.00], *p* = 0.94). The percentage of patients with each CHESS was as follows: 0, 16 patients (13.8%), 2, 29 patients (25.0%); 3, 8 patients (6.9%); 4, 15 patients (12.9%); 5, 34 patients (29.3%); 7, 14 patients (12.1%).

When patients were divided into 3 risk groups (Low risk: CHESS 0, 16 [13.8%] patients; Medium risk: CHESS 2–4, 52 [44.8%] patients; High risk: CHESS 5–7, 48 [41.4%] patients), the observed rates of embolic stroke for low‐, medium‐, and high‐risk group were 25.0%, 69.2%, and 89.6%, respectively, which were also well‐calibrated with the predicted rates. (Hosmer‐Lemeshow goodness‐of‐fit chi‐square = 0.4, *p* = 0.6, Figure 3B and Figure 4B).

### 3.3 Performance of CHESS with AF history excluded

When AF history was excluded, CHESS could also reliably predict embolic stroke with an AUC of 0.71 (95%CI 0.64–0.78) in the derivation cohort and an AUC of 0.65 (95%CI 0.55–0.75) in the validation cohort, both with satisfactory calibration (Derivation cohort: Hosmer‐Lemeshow goodness‐of‐fit chi‐square = 2.2, *p* = 0.1; Validation cohort: Hosmer‐Lemeshow goodness‐of‐fit chi‐square = 0.03, *p* = 0.9).

## 4 DISCUSSION

We developed an integer‐based score, CHESS, to identify embolic stroke preceding endovascular treatment in acute ischemic patients with LVO. The score was derived and validated in a multicenter‐based cohort of consecutive Chinese acute stroke patients with endovascular treatment. The proposed score assigns 2 or 3 points to each of the five objective factors: history of AF, 3 points; no history of hypertension, 2 points; DT>6 s/DT>3 s≥0.23, 2 points. According to predicted and observed probabilities, patients with a CHESS of 0 could be identified as low‐risk of embolic stroke, with a CHESS of 2–4 could be identified as medium‐risk and with a CHESS of 5–7 could be regarded as high‐risk.

Since endovascular treatment has become a routine practice for acute ischemic stroke patients with LVO and the treatment window has been extended,^23^ many clinical, imaging, and biochemical markers have been used to assess stroke severity, identify stroke etiology, optimize treatment effect and predict outcomes.^24^, ^25^, ^26^ To our knowledge, this is the first model combining objective clinical and imaging factors to predict embolic stroke with LVO prior to thrombectomy. Previous studies have proposed several imaging markers to predict embolic stroke, with the requirement of acute MRI and/or the need for highly experienced imaging readers.^8^, ^9^, ^10^, ^11^, ^12^, ^13^, ^14^, ^15^ Scores to predict presence of AF, like Score for the Targeting of Atrial Fibrillation (STAF)^27^ and LADS,^28^ may also be useful to detect cardiogenic embolism. However, the need of echocardiography makes them only applicable for secondary stroke prevention. Notably, most of the markers were developed to predict cardiogenic embolic stroke, while the true etiology of embolic LVO stroke is a combination of cardiogenic embolism (usually AF), artery‐to‐artery embolism from large vessel atherosclerosis and embolic stroke of undetermined source. There were some patients in our cohort with artery‐to‐artery embolism and embolic stroke of undetermined source etiology, ensuring the diversity of this study. Our cohorts also include occlusions both in intracranial and extracranial cervical arteries, and occlusions of anterior and posterior circulations, meaning that CHESS has a much greater generalizability compared with previous imaging markers and biomarkers.

In our derivation cohort, patients with embolic stroke were older, more frequently female, had lower baseline blood pressure and a more severely impaired collateral flow compared with non‐embolic stroke, which was in accordance with the previous studies.^8^, ^16^, ^29^ As for stroke severity, similar with other studies,^29^ a more severe clinical manifestation (higher baseline NIHSS) was found in embolic stroke patients compared with the non‐embolic ones. History of hypertension has also been recognized as a significant risk factor of ICAD‐related occlusions in a meta‐analysis of 1967 patients.^30^ Therefore, stroke patients without previous history of hypertension would be regarded prone to embolic stroke.

History of previously diagnosed AF is a dominant contributor of embolic stroke, which is not very easy to access under emergent settings in Chinese stroke centers. The diagnostic rate of AF in China ranges from 20% to 45% in other registries of patients underwent endovascular treatment.^3^, ^4^, ^31^, ^32^ Moreover, even for patients with a definite AF history, there is still a possibility of in‐situ thrombosis (due to large vessel atherosclerosis, etc.). The incidence of AF history in non‐embolic LVO stroke patients ranges from 12% to 25% according to the current and previous studies.^32^, ^33^ To be noted, CHESS could still reliably predict embolic stroke when AF history was excluded from the model. Therefore, for patients without AF history, CHESS can be a very useful tool to deduce stroke etiology. While for patients with previously diagnosed AF, CHESS could further help to discriminate whether this index event is embolic‐related.

The prognosis of embolic or non‐embolic stroke with LVO has not yet reached a consensus. Some studies reported that non‐embolic stroke had a lower reperfusion rate when using thrombectomy techniques only and tended to have a longer procedure time,^5^, ^33^, ^34^ leading to poor 3 month functional outcomes in patients with ICAD.^29^, ^33^ Thus, developing different recanalization strategy in atherosclerotic stroke patients to shorten the revascularization time is warranted. Moreover, Yang et al. from The Endovascular Treatment for Acute Anterior Circulation Ischemic Stroke Registry study in China showed that for acute patients with large vessel atherosclerosis, using angioplasty with balloon or stenting as first‐line therapy could enable more prompt revascularization, resulting in a better clinical outcome.^35^ Therefore, CHESS can arm stroke neurologists with a tool to identify non‐embolic stroke at hyperacute stage, and can be potentially useful to guide treatment strategy for neurointerventionalists, improving the outcomes of non‐embolic stroke patients.

There are some inevitable limitations of our study. First, the sample size is limited. However, consecutive and complete clinical and imaging data from 5 national comprehensive stroke centers have been included and the validation has proven the generalizability of this model. Second, around 27% patients were excluded due to incomplete imaging or clinical data, which might affect the generalizability of CHESS. However, since the performance of CHESS in the validation cohort was satisfactory, the generalizability could be warranted. Third, the percentage of embolic stroke is much higher than the non‐embolic ones in our cohorts, which might be due to the fact that many neurointerventionalists preferred to perform endovascular treatment for embolic (especially cardioembolic) stroke patients rather than non‐embolic (especially ICAD) patients, since embolic stroke patients have a higher recanalization rate as mentioned above. Fourth, the application of CT Perfusion in posterior circulation stroke has not been validated. But no difference was found between the AUCs of CHESS in LVO patients of anterior and posterior circulation. Fifth, emergency electrocardiograms were not collected for INSPIRE study. There were patients without known history of AF with an AF‐electrocardiogram implicating an embolic stroke. Sixth, previous medical history was provided by patients and relatives, which might not be accurate. Additionally, not all the possible risk factors were included in the initial screen, such as history of drinking and other atherosclerotic diseases. Nevertheless, the aim of this study was to derive a model that could identify stroke etiology as simple and as fast as possible under emergent settings. Seventh, though the inclusion criteria of the time window in this study is 24 h after onset, most of our patients are within the traditional 6 h time window. And the median age of our derivation cohort is 69 years old. Additionally, CHESS is derived and validated from a Chinese‐only population. Therefore, for patients in an extended time window (6–24 h) and with a younger age, and patients of other ethnic groups, CHESS should be applied with caution. Lastly, CHESS requires real‐time advanced post‐processing software of perfusion images to quantify collateral flow, which is not available in every stroke center and is not guideline‐recommended for patients within 6 h of stroke onset. Therefore, a score that uses magnetic resonance angiography (MRA)/CTA to semi‐quantify the level of collateral flow could probably be more practical. To be addressed, all the five centers of this research joined the INSPIRE study and acute multimodal CT scan has become a routine process after patients’ arrival at the ER, so no selection bias was considered to exist during patient selection. Since the concept of “tissue window” may replace the traditional “time window,” CHESS puts forward another possible application of perfusion CT in the individualized treatment of acute stroke.

## 5 CONCLUSIONS

CHESS, consists of history of AF, history of hypertension and quantified acute cerebral collateral flow, can reliably predict acute embolic stroke with LVO to assist reperfusion strategy in endovascular treatment.

## AUTHORS’ CONTRIBUTIONS

All author participated in data collection, critical review and revision of this manuscript. XC, MP, QD designed the study. LH, LL analyzed the data. LH drafted this manuscript, prepared tables and figures.

## DISCLOSURE

The authors have nothing to disclose.

## Supporting information

Table S1‐S2

## Data Availability

The data that support the findings of this study are available from Dr. Xin Cheng (chengxin@fudan.edu.cn) upon reasonable request.
