# Supplementary material for: Identification of embolic stroke in patients with large vessel occlusion: The Chinese embolic stroke score, CHESS
Source: CNS Neurosci Ther. 2021 Sep 24;28(4):531–9. doi: 10.1111/cns.13729 (PMC8928917; doi:10.1111/cns.13729)
Supplement: Supplementary file 1 — Table S1‐S2 [file CNS-28-531-s001.docx]

**SUPPLEMENTARY MATERIAL**

| Supplementary Table 1 CTP Acquisitions Protocols of 5 stroke centers | | | | |
| --- | --- | --- | --- | --- |
| Site^†^ | CT Scanner | Acquisitions | Contrast | Coverage |
| Site 1 | GE Medical Systems Discovery CT750 HD;  (64 detectors) | 26 acquisitions in 45 seconds; shuttle mode | 40 mL of contrast agent (Ultravist 370) injected at 4.5 mL/s | 80 mm |
| Site 2 | Siemens  SOMATOM Definition Flash (256 detectors) | 25 acquisitions in 62 second | 15 mL of contrast agent (Ultravist 370) injected at 4mL/s | 100mm |
| Site 3, Site 4, Site 5 | Toshiba Medical Imaging；Toshiba Aquilion ONE  (320 detectors) | 19 acquisitions in 65 seconds | 40 mL of contrast agent (Ultravist 370) injected at 6mL/s | 160mm |

^†^The name of each site was blinded for blind review.

| Supplementary Table 2 Baseline clinical profiles and CTP data^†^ | | | |
| --- | --- | --- | --- |
|  | Derivation Cohort (n=213) | Validation Cohort (n=116) | P |
| **Age,** median (IQR), yrs | 69.0 (60.5, 76.0) | 71.5 (62.3, 81.8) | 0.04 |
| **Male** | 128 (60.1%) | 71 (61.2%) | 0.84 |
| **Baseline SBP,** mean (SD), mmHg | 148.0 (24.1) | 147.2 (25.1) | 0.77 |
| **Baseline DBP,** mean (SD), mmHg | 83.0 (72.5, 92.0) | 80.0 (74.0, 91.0) | 0.65 |
| **Baseline NIHSS,** mean (SD) | 16.0 (12.0, 20.0) | 17.5 (8.0) | 0.79 |
| **Baseline Glucose,** median (IQR), mmol/L | 7.1 (6.3, 8.8) | 7.1 (6.0, 9.2) | 0.85 |
| **Medical History** |  |  |  |
| History of Smoking | 63 (29.6%) | 32 (27.6%) | 0.70 |
| History of Hypertension | 135 (63.4%) | 72 (62.1%) | 0.81 |
| History of Atrial Fibrillation | 112 (52.6%) | 56 (48.3%) | 0.46 |
| History of Dyslipidemia | 29 (13.6%) | 11 (9.5%) | 0.21 |
| History of Diabetes Mellitus | 39 (18.3%) | 30 (25.9%) | 0.11 |
| Past History of Stroke or TIA | 31 (14.6%) | 19 (16.4%) | 0.66 |
| **Embolic Stroke** | 153 (71.8%) | 83 (71.6%) | 0.96 |
| **Cause of Stroke** |  |  |  |
| Large Artery Atherosclerosis | 69 (32.4%) | 39 (33.6%) |  |
| Cardiac Embolism | 113 (53.1%) | 57 (49.1%) |  |
| Others^‡^ | 31 (14.6%) | 20 (17.3%) |  |
| **Intravenous Thrombolysis** | 112 (52.6%) | 49 (42.2%) | 0.07 |
| **Occlusion Site** |  |  | 0.94 |
| ICA | 51 (23.9%) | 26 (22.4%) |  |
| MCA-M1 | 113 (53.1%) | 59 (51.0%) |  |
| MCA-M2 | 8 (3.8%) | 5 (4.3%) |  |
| ICA+MCA-M1 | 13 (6.1%) | 10 (8.6%) |  |
| ACA | 1 (0.5%) | 1 (1.0%) |  |
| PCA/BA/VA | 27 (12.7%) | 15 (12.9%) |  |
| **Onset to door time,** median (IQR), min | 193.0 (109.5, 290.0) | 188.0 (83.0, 288.0) | 0.76 |
| **Infarct core,** median (IQR), mL | 18.0 (5.0, 43.0) | 15.0 (4.0, 40.0) | 0.33 |
| **Penumbra**, median (IQR), mL | 83.0 (55.1, 113.0) | 85.3 (50.0, 122.8) | 0.67 |
| **DT>3s**, median (IQR), mL | 113.0 (69.5, 153.0) | 110.5 (64.0, 157.3) | 0.93 |
| **DT>6s**, median (IQR), mL | 35.0 (9.0, 67.0) | 42.0 (9.0, 74.5) | 0.67 |
| **DT6/DT3 ratio**, median (IQR) | 0.3 (0.1, 0.5) | 0.4 (0.1, 0.5) | 0.49 |
| ^†^ Data are presented as number (percentage) of patients unless otherwise indicated  ^‡^ Other causes of stroke included dissection of ipsilateral carotid artery, embolic stroke of undetermined source and syphilis.  Abbreviations: IQR Interquartile range; SBP systolic blood pressure; DBP diastolic blood pressure; MAP mean arterial pressure; NIHSS National Institutes of Health Stroke Scale; TIA transient ischemic attack; ICA internal carotid artery; MCA-M1 M1 segment of middle cerebral artery; MCA-M2 M2 segment of middle cerebral artery; ACA anterior cerebral artery; PCA posterior cerebral artery; BA basilar artery; VA vertebral artery; DT delay time | | | |
